# Relationship between physical exercise, bullying, and being bullied among junior high school students: the multiple mediating effects of emotional management and interpersonal relationship distress

**DOI:** 10.1186/s12889-024-20012-y

**Published:** 2024-09-13

**Authors:** Qiang Zhang, Wenjing Deng

**Affiliations:** 1 Capital University of Physical Education and Sports, Beijing, China; 2 Chong Qing Yong Chuan Vocational Education Central School, Chong Qing, China

**Keywords:** Physical activity, Bullying, Being bullied, Emotion regulation self-efficacy, Interpersonal relationship distress, Chain mediating effect

## Abstract

**Objective:**

This paper investigates the relationships between physical activity (PA), school bullying, emotion regulation self-efficacy (ERS), and interpersonal relationship distress (IRD) among junior high school students. It also examines the underlying mechanisms of school bullying to provide insights into reducing adolescent bullying and to lay the groundwork for preventing and controlling aggressive behaviors.

**Methods:**

A survey was conducted on 484 students (240 males, 12.18 ± 0.8 years) from 4 secondary schools using the Physical Activity Rating Scale (PARS), Emotional Management Self-Efficacy Scale (EMSS), Interpersonal Relationship Distress Scale (IRDS), and Campus Bullying Scale (CBS) to examine the effects among the variables. A stratified random sampling method was used to select the sample, and data were collected with a structured questionnaire. The data were analyzed using SPSS 24.0 and AMOS 24.0 statistical software. The analysis included Pearson correlation analysis, structural equation modeling, and bias-corrected percentile Bootstrap methods.

**Results:**

(1) PA negatively predicts IRD, which in turn has an indirect effect on bullying (PA → IRD → Bullying), ES = -0.063. Additionally, EM and IRD act as mediators between PA and school bullying (PA → EM → IRD → Bullying), ES = 0.025. (2) PA negatively predicts IRD, which has an indirect effect on being bullied (PA → IRD → Being bullied), ES = -0.044. EM and IRD serve as chain mediators between PA and being bullied (PA → EM → IRD → Being bullied), ES = -0.071.

**Conclusion:**

PA can positively predict bullying, but it can be mitigated through EM to reduce IRD, thereby decreasing the occurrence of campus bullying and being bullied.

School bullying is considered an aggressive, intentional, and repetitive behavior, occurring without clear motivation, inflicted by one or more students on others. It not only causes physical harm to the victims but also negatively affects their mental health [1, 2]. School bullying is prevalent in some East Asian countries [3], having profound and lasting effects on the health and well-being of the victims [4, 5]. In children and adolescents, school bullying is closely associated with depression, anxiety, and insomnia [6]. Severe bullying can lead to self-harm [7]. Bullying peaks between the ages of 11 and 13, during the transition from primary to secondary school [8]. Given the potential harm of bullying to mental health, it is necessary to explore the mechanisms of bullying and victimization to provide a theoretical basis for preventing bullying among middle school students.

Physical activity (PA) has demonstrated significant mental health benefits, including strong anti-depressive and anti-anxiety effects, improved self-efficacy, and enhanced mood regulation [7, 9]. PA has also proven to be an effective intervention in anti-bullying programs for special populations with mental disorders, overweight, or obesity [10, 11]. As a vital component of public health strategies against bullying, PA positively influences the psychological well-being of both perpetrators and victims. Studies abroad have confirmed a close relationship between school bullying, victimization, and the frequency and type of PA. Students who engage in PA at least four times a week show higher aggression scores than those with lower exercise frequencies [9]. Studies suggest that regularly exercising adolescents are more likely to become bullies and exhibit higher aggression compared to their non-exercising peers [12]. Nikolaou’s study suggests that individuals who frequently participate in competitive sports are more likely to become bullies but are less likely to be victimized [13]. It recommends increasing opportunities for adolescents to exercise while enhancing supervision of exercise content and venues. Other studies confirm that physical education classes protect against bullying, with regularly exercising girls showing lower levels of victimization [14]. Waasdor used binary logistic regression to examine the relationship between health-related behaviors and bullying, finding that PA significantly reduces the likelihood of students becoming victims [15]. Pacífico et al. systematically reviewed the relationship between bullying victimization, aggressive behavior, and participation in physical activities and sedentary behaviors, finding that victims are associated with reduced PA and increased sedentary time [11]. Based on these findings, we hypothesize that H1a: PA can increase the likelihood of bullying behavior. H1b: PA can decrease the likelihood of being bullied.

Recent studies have found that emotion regulation self-efficacy (ERS) and interpersonal relationship distress (IRD) are crucial in preventing school bullying. Muris defines ERS as an individual’s perceived ability to manage negative emotions, including the belief in one’s capacity to avoid or recover from such states [16]. Effective ERS is essential for mental and physical health and is considered a protective factor against negative emotions. For instance, self-talk can help regain a positive attitude or calm oneself during fear and anxiety. Bandura, in his self-efficacy and social cognitive theories [17, 18], emphasizes that self-efficacy and self-regulation strategies are crucial for behavior change. Regular PA promotes both physical and psychological health, including a healthy lifestyle, body awareness, and confidence in physical skills. It also enhances safety, responsibility, patience, courage, and psychological balance [19]. Valois et al. surveyed over 3,800 students and found that PA is related to ERS [20]. Continuous PA can enhance self-efficacy, which, if not managed, can lead to decreased academic performance, social adaptation disorders, emotional depression, and an increased likelihood of deviant behavior. Moreover, regular physical activity offers multiple psychological benefits, such as enhanced self-control and self-esteem, both of which are closely linked to a decrease in bullying behaviors [21]. Participation in sports can mitigate the effects of bullying and is an effective strategy for promoting positive peer interactions and emotional regulation among adolescents.

IRD refers to the inability to establish and maintain meaningful relationships, lack of stable personal identity and self-awareness, and the use of avoidance strategies to manage strong emotions [22]. Méndez et al. found that poor relationships among students can result in bullying [9]. School bullying, a negative social interaction among adolescents, can be predicted by negative relationships with parents, teachers, and peers [23]. Gross’s emotion regulation self-efficacy theory emphasizes the impact of emotion management on social interactions and relationships [24]. PA can serve as an ERS strategy, improving emotion management through stress relief and emotional state enhancement, thereby improving interpersonal relationships.

In recent years, research has increasingly focused on how PA can improve adolescents’ emotion management and interpersonal relationships, thereby reducing bullying and victimization. González’s study confirmed that adolescents’ participation in group sports brings joy, improves poor interpersonal relationships, and promotes harmonious peer relationships [25]. Further research found that non-competitive physical activities convey values, promote prosocial attitudes, prevent bullying and victimization, and reduce the risk of aggressive incidents [26].

Based on these findings, we hypothesize H2a: PA can improve individuals’ ERS abilities, thereby reducing the occurrence of school bullying behaviors. H2b: PA can indirectly decrease the likelihood of individuals becoming victims of bullying by enhancing their ERS abilities. H3a: PA can alleviate IRD, thereby reducing the frequency of bullying behaviors. H3b: PA can further lower the risk of students being bullied by mitigating IRD.

According to Sullivan’s interpersonal theory [27], individuals seek interpersonal interactions during their adolescence. If they cannot effectively control their emotions, it may lead to psychological stress and social interaction difficulties [28]. Jun et al., in a cross-sectional survey study of 207 medical students, found that appropriate expression of anger can enhance their ERS ability, thereby improving interpersonal interaction skills [29]. ERS ability is a cognitive variable that influences behavioral and emotional processes [30]. Individuals with high levels of ERS ability believe they can achieve desired outcomes through their efforts, thus choosing effective coping strategies. Moreover, they exhibit more patience in the process of achieving their goals [30, 31]. Therefore, the ability to manage emotions may enhance individuals’ confidence, stimulate motivation for communication with others, and enable them to handle interpersonal relationships more effectively, avoiding disharmony in relationships.

Based on the above, the hypotheses are proposed: H4a: PA can enhance ERS, thereby alleviating IRD and preventing school bullying. H4b: PA can enhance ERS, thereby reducing IRD and decreasing the risk of being bullied.

This study employed PA as the independent variable, school bullying and being bullied as the dependent variables, and ERS and IRD as mediating variables to develop a chain mediation model (Fig. 1). The model aims to elucidate how PA reduces IRD through the enhancement of ERS, thereby preventing school bullying. This theoretical framework clarifies the research objectives and provides direction for subsequent analysis.

**Fig. 1:**
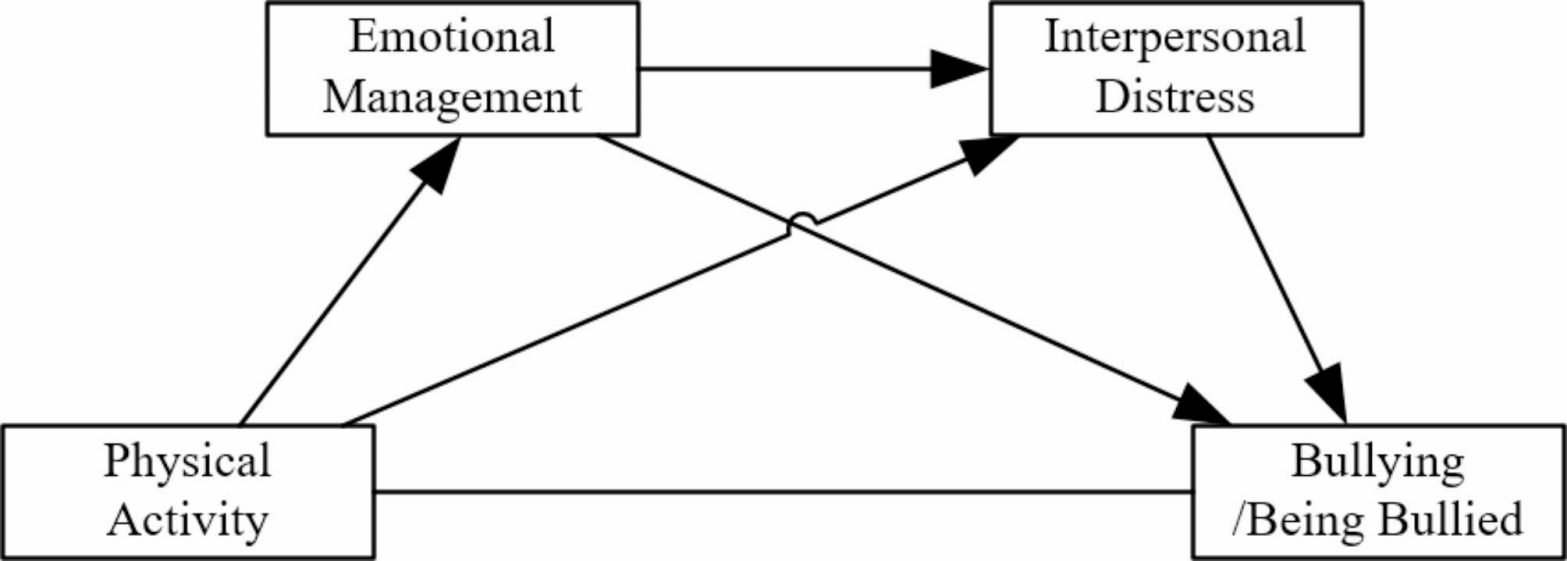
Multiple mediation model of school bullying

## Materials and methods

### Participants

From March to May 2023, we utilized a multi-stage cluster random sampling method to select two key junior high schools and two regular junior high schools in Shandong Province. Within each school, 1–2 classes from grades 6 through 9 were randomly chosen to participate in the survey, which was administered via the Wenjuanxing platform. Ultimately, 15 classes took part, and we issued 529 questionnaires, each taking an average of 8 min to complete. After excluding responses with repetitive patterns or completion times under 3 min, 45 invalid responses were discarded, resulting in 484 valid responses and a response rate of 91.5%. The sample included 240 male and 244 female students; 171 were only children, while 313 had siblings. The breakdown by grade was as follows: 87 students in grade 6, 137 in grade 7, 134 in grade 8, and 126 in grade 9. The average age of the participants was 12.18 years with a standard deviation of 0.8 years. Detailed demographic information is presented in Table 1.

**Table 1:** Basic information of the sample(*N* = 484)

Category		Frequency	Percentage(%)
Gender	Male	240	49.59
	Female	244	50.41
Grade	6^th^	87	17.98
	7^th^	137	28.31
	8^th^	134	27.69
	9^th^	126	26.03
School Characteristics	Key	261	53.93
	Regular	223	46.07
Child Status	Only	171	35.33
	Non-only	313	64.67

### Procedures

This study utilized a cross-sectional design and structured questionnaires to collect the necessary data. The questionnaires used in this study were revised in China, widely utilized, and demonstrated high reliability and validity. To further ensure their reliability and validity, we conducted additional reliability analysis and exploratory factor analysis. The study followed these procedures: Approval was first obtained from the Human Research Ethics Committee of Capital University of Physical Education and Sports. Afterward, researchers received consent from the principals of the selected schools and coordinated with grade-level directors to select the participating classes. Class teachers then distributed informed consent forms to students and their parents, explaining that participation was voluntary and confidentiality was assured. They also confirmed the number of participants. Finally, during physical education classes, teachers organized students to complete the questionnaires anonymously using the Wenjuanxing platform in the school information room. Researchers were present on-site to address any participant questions.

### Instruments

#### Physical activity rating scale (PARS)

The PARS revised by Liang (1994) [32], was employed to assess the PA levels of middle school students and investigate their exercise habits. This scale evaluates the intensity (e.g., “light exercise”), duration (e.g., “less than 10 minutes”), and frequency (e.g., “less than once a month”) of PA, with each dimension rated on a 5-point scale from 1 (low) to 5 (high). The amount of PA is calculated using the formula: PA = Intensity × Duration × Frequency. Both intensity and frequency are rated on a scale from 1 to 5, while duration is rated from 0 to 4. The possible scores range from 0 to 100 points. The activity levels are categorized as follows: 0–19 points indicate low activity, 20–42 points indicate moderate activity, and 43–100 points indicate high activity. In this study, Cronbach’s alpha for the scale was 0.807.

#### Emotion regulation self-efficacy scale (ERSS)

The ERSS, developed by Li [33], was used. This scale contains 17 items, covering four dimensions: Expressing Positive Emotions (EPM) with 4 items (e.g., “showing joy when something good happens”), Regulating Anger (AM) with 3 items, Regulating Depression (RD) with 4 items (e.g., “not feeling dejected when strongly criticized”), and Regulating Fear (RF) with 6 items (e.g., “not feeling scared in the dark”). In this study, Cronbach’s alpha for the total scale was 0.955, with the four dimensions being 0.911, 0.822, 0.907, and 0.889 respectively. The overall confirmatory factor analysis fit indices for the scale were: χ2/*df* = 5.03, CFI = 0.96, TLI = 0.92, RMSEA = 0.06, and SRMR = 0.05.

#### Interpersonal relationship distress scale (IRDS)

The IRDS developed by Deng & Zheng [34] was used. This questionnaire consists of 28 questions, with a binary response format (“Yes” or “No”). Higher scores indicate more severe IRD. The scale includes four dimensions, each with 7 items: Conversation Trouble (CT) (e.g., “finding it difficult to talk about personal troubles”), Interaction Trouble (IT) (e.g., “feeling uncomfortable when meeting strangers”), Trouble Treating Others (TTO) (e.g., “feeling excessive envy and jealousy towards others”), and Exposure to Heterosexual Distress (EHD) (e.g., “feeling unnatural when interacting with the opposite sex”). In this study, the Cronbach’s alpha for the questionnaire was 0.889. The confirmatory factor analysis fit indices were: χ2*/df* = 2.603, CFI = 0.89, TLI = 0.87, RMSEA = 0.05, and SRMR = 0.04.

#### Campus bullying scale (CBS)

The bullying subscale of the Olweus Bullying Questionnaire [35], revised by Zhang & Wu [36], consists of 12 items. It uses a 5-point Likert scale to measure the frequency of bullying behaviors. Six items assess bullying (BULLY) (e.g., “I spread rumors about some classmates to make others dislike them”), and six items assess victimization (VIC) (e.g., “others call me unpleasant nicknames, insult me, or mock me”). The frequency of occurrence is rated from 0 to 4, ranging from “never happened” to “several times a week”. In this study, the Cronbach’s alpha for the questionnaire was 0.843. Confirmatory factor analysis indicated good structural validity, with fit indices as follows: χ2/*df =* 2.94, CFI = 0.97, TLI = 0.98, RMSEA = 0.05, and SRMR = 0.03.

### Data analysis

The social statistical analysis software SPSS 24.0 was used for internal consistency testing and Pearson correlation analysis of PA, ERS, IRD, and school bullying. The AMOS 24.0 software was used for confirmatory factor analysis, mediation analysis, and Bootstrap analysis for difference testing. A significance level of α = 0.05 was set for statistical significance.

## Results

### Control and testing for common method bias

All data in this study were self-reported by adolescents, which may be affected by common method bias. Therefore, in the study design and data collection process, measures were taken such as making the questionnaire anonymous, separating different questionnaires, reverse scoring some items, and emphasizing the confidentiality of the data for pre-program control. In addition, confirmatory factor analysis was used to test for common method bias in all self-reported items. The results showed a poor model fit, with χ2/*df* = 29.44, CFI = 0.47, GFI = 0.55, AGFI = 0.39, NFI = 0.46, and RMSEA = 0.24. This indicates that there is no serious common method bias issue in this study.

### Means, standard deviations, and correlation analysis of variables

Descriptive statistics and correlation analysis (Table 2) reveal significant relationships: gender is significantly related to PA and interpersonal relationships (*r*=-0.272, *P* < 0.01; *r* = 0.107, *P* < 0.05). ERS is significantly related to bullying and IRD (*r*=-0.134, *P*<0.01) (*r*=-0.316, *P*<0.01), and ERS is positively correlated with PA (*r* = 0.161, *P*<0.01). IRD is negatively correlated with PA (*r*=-0.132, *P*<0.01), and positively correlated with bullying and being bullied (*r* = 0.306, *P*<0.01) (*r* = 0.207, *P*<0.01). Given that the relationship between gender and bullying was not significant, subsequent analyses did not differentiate between male and female students.

**Table 2:** Descriptive statistics of variables and correlation matrix among variables (*N* = 484)

Variable	Mean	Std. Dev.	1	2	3	4	5	6
Gender	-	-	1					
PA	28.7	19.78	-0.272	1				
ERS	74.29	21.9	-0.05	0.161**	1			
IRD	5.84	5.45	0.107*	− 0.132**	− 0.316**	1		
Bullying	1.13	2.74	0.006	0.037	− 0.134**	0.306**	1	
Being Bullied	0.24	1.45	-0.034	0.014	-0.082	0.207**	0.534**	1

Significance:0.000: ***, *p* < 0.001; 0.001: **, *p* < 0.01;0.05: *, *p* < 0.05

### Chain mediating mechanism analysis between PA and school bullying

**Fig. 2:**
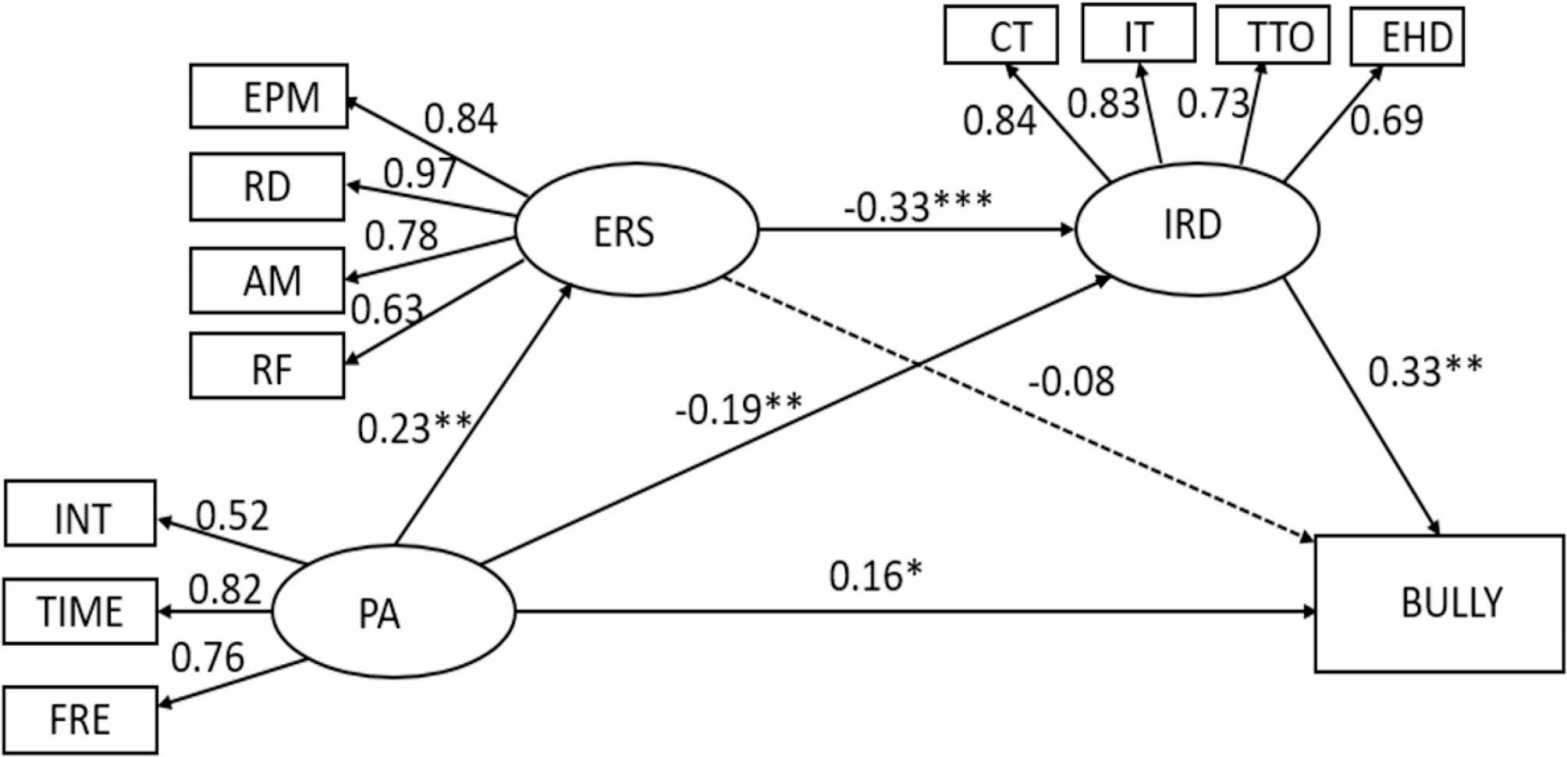
Chain mediations between PA and bullying. Note: BULLY = school bullying. Dash lines indicate an insignificant relationship

To effectively control measurement errors, this study used structural equation modeling to test for multiple mediating effects. First, based on the hypothesized model, PA was used as the predictor variable, bullying as the outcome variable, and ERS and IRD as mediating variables for path analysis. Figure 2 presents the data fit results: χ2/*df* = 3.001, CFI = 0.96, GFI = 0.953, RMSEA = 0.064, and SRMR = 0.041. All fit indices fall within acceptable ranges, confirming the validity of the initially proposed model.

The path coefficients and significance levels are illustrated in the diagram. PA significantly affects ERS and IRD, with standardized path coefficients of 0.23 and − 0.19, respectively. ERS negatively impacts IRD, with a standardized path coefficient of -0.33. IRD positively affects bullying, with a standardized path coefficient of 0.33. Additionally, PA positively affects bullying, with a standardized path coefficient of 0.16. However, ERS does not significantly impact bullying.

This study used the Bootstrap procedure to test the significance of the mediating effects, drawing 5000 samples with a 95% confidence interval. A mediating effect is considered significant if the 95% confidence interval for the path coefficients does not include 0. According to the results in Table 3, the path from PA to bullying is significant (*P* = 0.025), supporting hypothesis H1a. In contrast, the path from ERS to bullying is not significant (*P* = 0.103), which does not support hypothesis H2a.

Following Wen et al. [37], the interpretation of mediating effects depends on the signs of ab and c’. If ab and c’ have the same sign, the mediating effect is considered valid. This study found that both IRD and ERS act as suppressor variables in the relationship between PA and school bullying among adolescents: (1) PA reduces IRD, which indirectly decreases bullying (PA → IRD → Bullying), with a suppressor effect value of -0.063 (95% CI: -0.179, -0.028), supporting hypothesis H3a. (2) ERS and IRD also act as suppressors in the relationship between PA and school bullying (PA → ERS → IRD → Bullying), with a suppressor effect value of 0.025 (95% CI: -0.375, -0.037), supporting hypothesis H4a.

**Table 3:** Relationships of emotional management and interpersonal distress between PA and bullying

Predictor	Standardized Coefficient	Unstandardized Coefficient	Standard Errors.	Critical Ratios	*P*
PA → ERS	0.23	3.37	1.21	2.79	0.005
ERS → IRD	-0.33	-0.13	0.02	-6.12	< 0.001
PA → IRD	-0.19	-1.11	0.45	-2.47	0.014
EM → Bullying	-0.08	-0.06	0.04	-1.63	0.103
IRD → Bullying	0.33	0.64	0.1	6.16	< 0.001
PA → Bullying	0.16	1.82	0.81	2.24	0.025

### Path analysis of the chain mediation mechanism of PA and school bullying

**Fig. 3:**
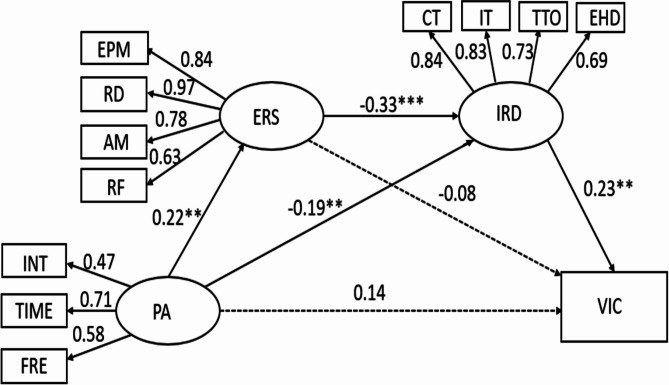
Chain mediations between PA and being bullied. Note: VIC = being bullied. Dash lines indicate an insignificant relationship

In this analysis, PA is treated as the predictor variable, and being bullied is the outcome variable. ERS and IRD serve as mediating variables. The model’s fit indices are within acceptable ranges: χ2*/df* = 2.704, CFI = 0.965, GFI = 0.957, RMSEA = 0.059, and SRMR = 0.039, as illustrated in Fig. 3. These results support the validity of the initially proposed model.

The figure illustrates the path coefficients and significance levels. PA significantly impacts ERS and IRD, with standardized path coefficients of 0.22 and − 0.19, respectively. ERS significantly negatively impacts IRD, with a standardized path coefficient of -0.33. IRD significantly positively affects being bullied, with a standardized path coefficient of 0.23. However, the effects of ERS and PA on being bullied are not significant.

Table 4 reveals that the paths from PA and ERS to being bullied are not significant (*P* = 0.054, 0.445), thus hypotheses H1b and H2b are not supported. The lack of a significant direct effect from PA to being bullied indicates that IRD and ERS fully mediate this relationship. This complete mediation consists of two pathways: (1) PA negatively affects IRD, which then indirectly impacts being bullied (PA → IRD → Being bullied), with a mediation effect of -0.044 (95% CI: -0.179, -0.028), supporting hypothesis H3b; (2) ERS and IRD mediate the relationship between PA and being bullied in a chain mediation model (PA → ERS → IRD → Being bullied), with a mediation effect of -0.071 (95% CI: -0.375, -0.037), supporting hypothesis H4b.

**Table 4:** Summary of model relationships of emotional management and interpersonal distress between PA and bullying

Predictor	Standardized Coefficient	Unstandardized Coefficient	S.E.	C.*R*.	*P*
PA → ERS	0.22	3.41	1.23	2.77	0.006
ERS → IRD	-0.33	-0.13	0.02	-6.18	< 0.001
PA → IRD	-0.19	-1.15	0.46	-2.49	0.013
ERS → Bullying	-0.04	-0.02	0.02	-0.76	0.445
IRD → Bullying	0.23	0.23	0.06	4.14	< 0.001
PA → Bullying	0.14	0.82	0.43	1.93	0.054

## Discussion

This study examines the predictive effects of PA on bullying and victimization, as well as the role of emotional management and interpersonal relationship issues in mediating this relationship. It reveals the mechanism by which PA predicts bullying and victimization through its influence on emotional management and interpersonal relationships.

### Direct effects analysis of PA on bullying and being bullied

The study found that PA can positively predict school bullying, but its predictive effect on victimization is not significant. Empirical studies on the relationship between PA and bullying/victimization in China are relatively scarce. The results of this study are consistent with most related studies both domestically and internationally, indicating that higher levels of PA may be associated with the occurrence of school bullying. Upon entering middle school, adolescents face the challenge of re-establishing peer relationships. During this period, active physical activities may lead to more frequent participation in various social activities on campus, thereby increasing the risk of exposure to potential conflicts. Without adequate supervision, these conflicts may escalate into bullying behaviors. However, some studies do not distinguish between bullying and victimization, suggesting that the higher the level of physical participation, the less frequent the occurrence of school bullying. These studies suggest that physical participation can enhance cognitive functions, reduce sensitivity to hostile information, and decrease attention to dangerous behaviors. Haney Aguirre-Loaiza et al. first confirmed through experimental intervention the positive effects of PA on inhibitory control and emotional situation recognition [38]. Additionally, physical participation can improve the quality of peer relationships, further reducing the occurrence of school bullying. PA may also enhance the cognitive flexibility and emotional regulation abilities of victims, helping them better cope with the negative impacts of bullying experiences. This study found that the direct predictive effect of PA on bullying may be related to the personality changes of adolescents during puberty and the high level of activity brought by PA. In this context, although the positive effects of PA may not be as significant as expected, it still helps in understanding the role of PA in school bullying.

For victimization, this study did not find a significant direct effect between PA and being bullied, which is consistent with the findings of Ortega [39]. Some studies suggest that regular participation in PA can reduce the likelihood of becoming a victim of bullying. PA is not only an important way to convey values but also enhances communication skills and promotes prosocial attitudes. Therefore, students’ physical activities are considered a health-promoting practice, and physically active students are generally believed to be more capable of protecting themselves. Hermoso and his team explored the relationship between PA, sedentary behavior, and the experience of bullying among children and adolescents [40]. They found that not meeting PA guidelines and excessive sedentary behavior are risk factors for being bullied, while at least 60 min of moderate-to-vigorous PA per day leads to better health and quality of life. Previous cross-sectional studies have also shown that not meeting these PA guidelines significantly increases the risk of bullying among children and adolescents. Therefore, PA is seen as an effective tool for preventing and reducing the occurrence of bullying. Hermoso et al. believe that students lacking PA are more likely to be bullied due to factors such as insufficient motor skills, poor physical fitness, and lack of confidence in participating in physical activities [40]. Nevertheless, this study did not find a significant direct effect between PA and being bullied. The occurrence of school bullying is influenced by various factors, including family background, cultural adaptation, bullying experience, and parental educational background. For example, Jang et al. pointed out that long-term bullying victimization is a potential risk factor for the mental health of children from multicultural families, particularly among adolescents whose mothers are from Southeast Asia [58]. The study by Kim and Fong explored the relationship between the bullying victimization experiences of children from multicultural families and their cultural adaptation and life satisfaction [41]. They found that entering bullying victimization is associated with reduced emotional cultural adaptation, and both entering and exiting bullying victimization are related to life satisfaction. Park used an asymmetric fixed effects model to evaluate the effects of entering and exiting bullying victimization [42]. He found that the mother’s college education level enhances the psychological health benefits of exiting bullying victimization but does not mitigate the harmful effects of entering it. The protective effect of the mother’s college education level is particularly significant for girls.

In summary, the direct predictive effects of PA on school bullying and victimization have not yet reached a consensus. Due to the complexity of the direct predictive effects of PA on bullying and victimization, more research is needed to further confirm this relationship and consider other potential influencing factors.

### Indirect effects Analysis of ERS and IRD

PA has a significant positive predictive effect on emotional management, consistent with most related studies [43]. Regular and sustained PA plays a crucial role in regulating emotions. When individuals are in an uncomfortable state, excellent emotional regulation abilities often lead to a reevaluation of existing cognitive elements related to interpersonal perception, memory, and thinking. This approach helps quickly find flexible and effective ways to avoid conflicts and contradictions in interpersonal interactions, aiding individuals in better integrating into groups [44].

According to the general aggression model, negative emotions such as anxiety, depression, and anger can bring negative experiences to adolescents, who may bully others to vent these unpleasant feelings [45]. However, this study did not confirm the hypothesis that emotional management negatively predicts bullying and victimization. Additionally, the effects of emotional regulation strategies vary in different contexts, and individuals can flexibly deploy these strategies according to changing situational demands [46]. Emotional regulation encompasses various strategies, such as cognitive reappraisal and emotional suppression. Individuals may use different strategies to cope with emotions, but this study did not distinguish between them, potentially obscuring the predictive role of emotional regulation self-efficacy on bullying and victimization. Additionally, differences in emotional regulation abilities among individuals and the ways male and female students handle positive and negative emotions may contribute to the non-significant relationship between emotional management and bullying/victimization.

The hypothesis that PA negatively predicts interpersonal relationship issues was confirmed in this study. Regular participation in PA increases opportunities for interaction among students, especially in group activities [47]. It promotes mutual interactions while reducing the occurrence of some negative emotions. Adolescents usually do not prefer to communicate with guardians such as family and teachers, leading to relatively less social support and help, which weakens their interpersonal communication skills during this period. When conflicts and disputes arise among students, they tend to use simple and rough methods to cope, leading to deteriorating peer relationships and escalating conflicts, which may trigger school bullying. The conclusion that interpersonal relationship issues positively predict school bullying and victimization was also confirmed in this study, consistent with most research results [25, 47, 48]. Negative interpersonal relationships among adolescents can directly predict aggressive behaviors. Previous studies have shown that poor conflict management skills in interpersonal relationships are a risk factor for bullying [49]. Longitudinal studies also show that reducing negative emotions like anxiety and depression and fostering positive peer perceptions can predict a reduction in victimization [50]. Adolescents with interpersonal relationship issues often experience frustration in real-life interactions. The widespread use of the internet and the development of mobile media lead them to escape reality, feel alienated from society, and seek fulfillment in the virtual world [51]. Adolescents during this period may view bullying as a reasonable way to solve problems and may become bullies in certain situations. Meanwhile, the excessive use of mobile phones, the internet, and other media takes up a lot of students’ time, making those who lack PA and are sedentary more likely to become victims of bullying [15].

This study also found that emotional management negatively predicts interpersonal relationship issues, similar to most domestic and international studies [52–54]. Liu proposed that emotional self-management ability, psychological resilience, and adolescent PA mutually promote each other, enhancing adolescents’ social communication skills [52]. With good emotional management strategies, individuals can transform negative emotions, stressful events, and disharmony into conditions that motivate self-development, reduce the experience of negative emotions, and handle interpersonal relationships more rationally, effectively alleviating awkwardness and discomfort in interactions [53]. Emotional management is closely related to the development of individual social cognitive characteristics and social communication skills [54]. Adolescents who frequently exhibit negative emotions may experience social withdrawal, lack of activity, and a lack of sports skills, leading to lower acceptance among peers, such as being less talkative or not fitting in. In contrast, good emotional management can help adolescents better handle various issues related to self-development and social interactions.

In summary, this study confirmed the positive predictive effect of PA on emotional management and its negative predictive effect on alleviating interpersonal relationship issues. This provides important guidance for the application of PA in preventing school bullying. By incorporating the challenges and stress of activity into daily physical education classes, students can learn and experience the effectiveness of emotional regulation during physical activities. Some studies have shown that participating in relaxation activities such as yoga [55] and meditation [56] can significantly promote emotional stability and enhance self-regulation abilities, helping students better control and adjust their emotions during exercise, thus more effectively coping with school pressures and conflicts. Additionally, encouraging participation in group activities [57] can significantly enhance students’ social skills, increase peer interactions, and reduce feelings of isolation and interpersonal conflicts. Implementing these strategies can help schools effectively reduce the impact of interpersonal relationship issues on bullying and victimization, and improve students’ social environment. Ultimately, this will help create a positive and healthy living environment for students, promoting their overall well-being and healthy development.

## Strengths and limitations

This study, grounded in the theories of self-efficacy and interpersonal relationships, incorporates ERS and IRD as mediating variables. It separately models and explores the relationships and underlying mechanisms between physical activity (PA), bullying behavior, and being bullied. The findings provide empirical evidence and intervention recommendations for addressing school bullying, assisting educators and society in effectively tackling issues that impact students’ physical and mental health.

However, this study has some limitations. Firstly, as a cross-sectional study, it cannot infer causal relationships between variables. Future research could employ longitudinal tracking or experimental intervention designs to better explain the impact of PA on school bullying. Secondly, while this study considered the mediating roles of self-efficacy and IRD, other potential moderating factors such as cultural adaptation [41] and parental education level [58] might also influence the research outcomes. Future research could incorporate these factors to achieve a more comprehensive understanding of the complex relationship between bullying victimization and mental health. Additionally, the study’s participants were junior high school students from Eastern China, which may limit the generalizability of the findings. Future research should expand the sample to include students from more countries and regions. Lastly, this study explored the predictive mechanism of bullying based on students’ PA levels, but the practical implications of the findings need further strengthening. Future research should examine the effects of different forms, intensities, and types of PA on school bullying.

## Conclusion

In the model predicting school bullying, PA significantly predicts bullying, ERS, and IRD. ERS negatively predicts IRD but does not significantly predict bullying. Additionally, IRD significantly predicts bullying. The prediction of school bullying by PA includes masking effects, with two primary pathways: (1) PA → IRD → bullying, and (2) PA → ERS → IRD → bullying. In the model predicting being bullied at school, PA does not significantly predict this outcome. However, PA significantly positively predicts ERS and negatively predicts IRD. ERS significantly negatively predicts IRD but does not significantly predict being bullied. IRD significantly predicts being bullied. PA fully mediates the prediction of being bullied, with the pathways being: (1) PA → IRD → being bullied, and (2) PA → ERS → IRD → being bullied.

Based on the results of this study, we conclude that PA is an effective way to improve students’ emotional regulation and interpersonal relationships, significantly reducing both bullying and victimization. Given the prevalence of school bullying and its negative impact on mental health, public health policies should prioritize increasing adolescent participation in moderate-to-vigorous physical activity. This not only enhances physical health but also effectively improves emotional regulation and develops students’ social interaction skills. Additionally, it is recommended that structured physical activity be incorporated into school curricula to create a relaxed and enjoyable learning environment, improving peer relationships and maximizing the prevention of school bullying.

## Data Availability

The datasets of this study are available from the corresponding author on reasonable request.

## Abbreviations

CBS: Campus Bullying Scale
CFI: Comparative Fit Index
CT: Conversation Trouble
EMSS: Emotional Management Self-Efficacy Scale
ERS: Emotion Regulation Self-Efficacy
EPM: Expressing Positive Emotions
EHD: Exposure to Heterosexual Distress
GFI: Goodness of Fit Index
IRDS: Interpersonal Relationship Distress Scale
IRD: Interpersonal Relationship Distress
IT: Interaction Trouble
MVPA: Moderate-to-Vigorous Physical Activity
PA: Physical Activity
PARS: Physical Activity Rating Scale
RMSEA: Root Mean Square Error of Approximation
RF: Regulating Fear
RS: Regulating Stress
SRMR: Standardized Root Mean Square Residual
TLI: Tucker-Lewis Index
TTO: Trouble Treating Others
VIC: Victimization
